# ConsenTrack—Blockchain Based Framework for Open Banking Consent Data Tracking

**DOI:** 10.1007/s44230-023-00023-5

**Published:** 2023-04-12

**Authors:** Abir Ghosh, Indraneel Mukhopadhyay, Subhalaxmi Chakraborty

**Affiliations:** 1 University of Engineering and Management, Kolkata, West Bengal India; 2 Computer Science & Engineering, Institute of Engineering and Management, Kolkata, West Bengal India; 3 Department of Computer Science, University of Engineering and Management, Kolkata, West Bengal India

**Keywords:** Blockchain, Open banking, Consent Management, Bank, Regulator, Customer Trust, Data Violation

## Abstract

Consent management is most critical part of open banking. Customers, banks, third party service providers, regulators are various parties involved into this process. The recent data shows that open banking has not been greatly accepted yet by customers to the fullest capability. Recent surveys conducted on usage of open banking indicates the discomfort in customer mind about data sharing. Blockchain based framework implementation can bring the required transparency into the consent management process. To achieve that Blockchain technology needs to be embraced by banks and Third party providers (TPPs) to provide customers the open banking services in transparent manner. A blockchain based framework which can be easily integrated into banks’ existing technology landscape thus becomes need of the hour. Consortium permissioned blockchain based framework implemented in Corda is suggested in this paper which addresses challenges faced by customers and it tracks data sharing violation for communicating to customers. Data sharing between bank and TPPs happen as node to node transaction and regulatory bodies can have tracking of every such transactions as owner of Notary node. Based on the legal contract between bank and TPP, framework compares and finds out in real time if any data sharing violation happening. Real-time tracking of data sharing violation and communication to customer provides transparency into the framework which will boost customer confidence and trust into the system. Regulatory bodies need to actively own this part to share information with customers about the data handling if there is any violation**.**

## Introduction

In spite of push from central bank and regulators, customers are not providing consents for their data sharing in great numbers which is the most essential part for success of open banking. In UK, As of December 2020, there were 109 firms with live-to-market open banking-enabled products and services. Open banking is regulator initiative so there are timelines for the banks to adopt it. It is both an opportunity and threat for any financial organization which has started providing open banking service [1]. Opportunity in the sense that monetization of APIs can be done, customers can be retained by fulfilling their need of service at lower cost. Threat from other perspective where customers can go away if the services offered to customers are not competitive in the market and there are fear of monetary fine from regulator for missing timelines. Open banking also poses great responsibility to financial organization to handle customer data and customer consent in most transparent and secure manner as it is most critical area to earn trust of customers. As clearly stated in General Data Protection Regulation (GDPR) in Official Journal of the European Union [2], personal data of user (banking customers) cannot be used for processing purpose without taking consent from customer where data will be used for providing goods or services. It requires data subjects to control their personal data effectively- being informed about it and handle it in an intelligible manner [3].Thus it is a regulatory requirement for service providers to manage customer consents. It will lead to legal violations when service provider does not take user consent to use personal information of users.

### Issues Faced by Customer in Open Banking Consent Management

As stated by Open Banking UK organization, Open banking is enabling a world of innovative apps and services tailored to users’ financial data. It will benefit customers where they will have plenty of options which will bring down the cost of service. The revised Payment Services Directive 2 (PSD 2) by European Union targets to open market for payment related services where it will simulate competition in the market and it will benefit customer to look out for better services at the same or lower cost [4]. In case of open banking, it is all about payment and account information which makes it distinct as impact will be directly in the financial matter if not handled legitimate way. Customers are supported with regulations which enable them for making decisions on how to manage their own data. Even well-informed and rational customers cannot manage the issue of their personal data falling into undesired hand [5]. A survey was conducted to study perceptions and fears of open banking among digital naive women. Suela et al. [6] found that most of the consumers are against their data being shared with an organization unrelated to their banks. Bashir et al. [7] discussed that voluntariness and insufficient understanding of the whole exists in providing the informed consent to the management of customer personal information. If the data is to be shared with any unknown or little known organizations, customer often feels the hesitance whether to share data or not. Athapaththu [8] highlights that Customer can accept or deny if customer does not trust the requesting organization. Not much exploration happened so far how the consent management related issues faced by customers can be removed. Customer knowledge, customer psychology and customer economic condition etc. there are several factors which impacts customer decision making.

### Challenges Faced by Banks, TPPs and Regulators in Managing Customer Consent in Open Banking

Managing user consents effectively and efficiently brings lot of advantages to the service providers. Shafiq [9] explained it in elaborating consent management that some of these advantages are: protection against the data breaches, building trust and being compliant to regulations. Hence consent management is required by every party involved in the open banking space for their own benefit- be it customer, be it service providers or be it regulators. Though Open banking is regulator initiative, regulators does not have real-time view of customer data sharing between data custodian bank and TPP. Existing open banking framework does not have the capability to share real-time view of data sharing with regulator. This prevents regulators to take prompt action against defaulters as well as makes it difficult to keep customer informed about illegitimate data sharing. It is quite bit of challenging to make it mandatory keeping regulator involved in every case of open banking consent related transaction and data sharing transaction between bank and TPPs. Worldwide open banking adoption is progressing in different pace depending on how regulatory bodies of different countries are pushing for it. In many countries, open banking is not flourished yet fully and regulations are being framed to support adoption. Technology upgrade is not always easy for these TPP organizations. TPPs are mostly driven by innovation and their USP in most of the cases are cost effective products or services. Stiff competition in the market does not allow them to impact the product and service offering cost to customer. Regulators are bound to take care in making regulations which will make the open banking system easy to operate for the TPPs. Otherwise the main objective of open banking will not be achieved.

## Importance to have Transparency in Data Sharing

Multiple data tampering incidents and security incidents are there where traditional technologies could not play the role of enabler of trust [10]. Babin and Smith [11] highlights the need to have security and protections provided to consumers through a consistent framework when implementing open banking model. As stated in the ERI Open Banking white paper [12], having transparency in the data, processes and policies while maintaining data security is the key to success. Remolina [13] raised the concern that in case of open banking, the new structure for financial intermediation has positive and negative externalities that regulators should take into account when promoting or regulating open banking. Enrico and Roger [14] found from survey result that roughly seven out of ten people are concerned about their information being used for a different purpose from the one it was collected for. Polasik and Kotkowski [15] analyze the factors influencing adoption of open banking services in a pan-European survey and highlight that the preference for anonymity and reluctance to share data negatively impact the propensity to take to open banking, as well as the distrust of non-bank providers. Kirsten [16] mentions that with information misuse as a particularly salient form of risk online, respecting privacy is often closely tied to trust in consumer surveys. Rajaretnam [17] highlighted the same for e-commerce that consumers are concerned over the safety of their personal information and the violation of their privacy rights which can be described as being the single overwhelming barrier to rapid growth of e-commerce. On the similar note, Vikram et al. [18] elaborates that clear rules and effective consent on use and processing of data are necessary for an efficient and equitable data economy. Steve [19] provided stress on the fact that not just the opportunities, open banking brings lots of challenges also like privacy disclosure issues, data leakages issues, identity theft issues. Consent management process in open banking needs to address all these salient challenges like customer fear, customer trust factor, handling customer data secure way without improper data sharing, customer comfort, keeping customer informed about data sharing and address all these challenges properly. Transparency will provide confidence into customer mind. Transparency will add confidence into regulatory bodies. Transparency can be stated as the main pillar of success of consent management and open banking. It is one such important factor which needs to be maintained consistently.

## Why Existing Open banking Framework can not Provide Transparent Consent Management and Data Sharing

Babin and Smith [11] highlights that balance of activity and involvement between government and the private sector is a key question for open banking implementation. Unbalanced open banking model has a higher chance of failure. Organizations must adopt practices which help bringing transparency into the open banking process on a continuous basis. Post Covid19 when the processes have digitized more and more, it has become necessity to keep customer informed and to have processes as transparent as possible. Banks and TPPs need to make changes in their technology landscape which will allow them to keep customer and regulator informed in every case of consent related transaction or data sharing transaction. Associated cost and IT strategy along with prioritization makes it difficult for banks and TPPs to make necessary upgradations in their technology landscapes.

Identifying this gap of what existing technology landscape of banks and TPPs cannot do with respect to implementing a transparent data sharing process in open banking, we have done literature review on existing implementation approaches. Our literature review mainly consisted customer concern in open banking consent process and the use of blockchain implementation in the open banking area in the broader perspective.

## Literature Review

### Regulatory Aspect

With respect to GDPR compliances, Haque et al. [20] identifies that consent management, data subjects' right are less explored articles which needs more research focus. Accenture [21] report highlights it that by not allowing access to more information than absolutely necessary or than the user has consented to will avoid non-compliance to GDPR. Emma [22] discusses that the regulatory body which governs the market, bear the risk of losing trust of customer if customer data is not handled legitimate way. Role of governments in regulating and legislating will have more and more importance. Legislation requirement need to be suitable for an open banking paradigm and need to bolster customers control over his/her personal data. Regulatory bodies need to have control, visibility and control measures to handle data sharing proactively with efficiency rather handling it as the situation need is. Integrity (fairness), competence (ability/expertise), customer orientation (benevolence) and transparency (communications) are significant determinants of trustworthiness of financial services.

### Dilemma by Customer in Providing Consent

Since open banking is relatively new concept to digitally naïve customers, it becomes a bottleneck into the whole concept. Customer education on open banking will happen slowly. To gain the full potential of open banking, banks or organizations need to think about the unconventional ways to make customer aware about open banking concepts and specifically consent management concepts. To site an example, a study by the Unlimited Group identified that in 2017, Open banking was not known to 91% of UK bank customers.

To effectively manage customer consents, it is very critical to have trust of customers in the entire process so that customer provides consent for data sharing. Nesrin et al. [23] discussed that good and firm relation influence customer satisfaction in banking sector which in turn influence building trust which is the case in North Cyprus. Same will be applicable for other part of world also. Building trust is essential for customer consent management. Raija [24] highlights that degree of consumer trust varies depending on the service customer is looking at. It is highest in traditional bank accounts and it is lowest in investments and pensions. It will vary at the organization level, service level and even at the country-level. Spencer et al. [25] highlights that the amount of breached information is projected to be double from two to four billion items within next five years. This humongous uncontrolled dissemination of personal identities is a matter of concern about privacy.

### Importance of Informed and Transparent Consent

Edgar and Roser [26] published a detailed survey report and highlighted that consent provided to TPPs by the open banking customers are not informed consent. It depends on relationship between the discloser and the recipient if personal information can be shared with an online company. Individual needs to be clear how information will be used which will build a level of trust [27, 28]. It is proved by research findings that privacy concerns and the effect of intention to share personal information was mediated by trust [29, 30]. During Covid-19, banking has become more and more digital in nature. Everything offered to customers on banking sites, are needed more simplified now than ever. For an emerging economy like India, survey shows that discomfort negatively contributes to perceived ease of use and perceived usefulness. This survey was done to examine the use intention of open banking [31]. A simple process can make customer confident what they are doing with their money and with their data. Lee [32] highlights data will never be worth more than the confidence consumers have in an organization’s data practices. This paradox of trust have been explained by using several hypothesis. Like simplified banking processes, it is the transparency which make customers loyal to any banking organization. In the case of consent management process of open banking, simplicity and transparency are absolutely essential to keep the customer opting for open banking initiatives on continuous basis. Primary objective of open banking is to allow fin tech organizations to access customer data and provide services. Scott [33] analyses that customer could suffer more harm than gaining benefit from any open banking framework if customer data move from higher security environment to lower security environment where customer has not authorized data holder. Daiy et al. [34] states the need to have a model to check relative importance of banks’ crucial factors to select open banking strategic partners, which provide managerial insights and valuable guidance for the banking sector. It highlights the need of important scrutiny in selecting the partners who become an essential part of the open banking eco-system. In majority of cases, these fintech organizations are not under stricter surveillance of regulatory organizations, which makes the customers less confident about data sharing. It may be the technical limitations sometimes; third party organizations or banks cannot filter out unnecessary data of customers to be conforming to the GDPR like rules and regulations [35]. Integrity is the most important determinant here [36, 37]. Data leakage causes serious issues to organization. It causes not just the hefty financial fine but also irreversible reputational damage. In the current context when data breaches happening more frequently, detecting and preventing data loss has become most pressing security concern [38]. Mukhopadhyay and Ghosh [39] highlights the need of a framework involving all parties to identify data violations.

## Related Work

Various approaches taken by researchers worldwide to manage customer consent issues and challenges for open banking and other industries. Some of these are mentioned here which are related to our research area.

O-Consent [40] provides a protocol for lifecycle management of the consent for end user, business and organizations. It manages consent lifecycle within Permissionless local sidechain. It provides multiple authoritative proofs for consent receipt. It implements trusted timestamp proof in case of establishing validity of a signed consent agreement. In another approach [41], it proposes a data privacy management framework based on blockchain technology for the financial sector. It consists of three components: a data privacy classification method, a collaborative-filtering-based model and a confirmation data disclosure scheme for customer strategies based on the Nudge theory. It uses data classification method and customer data disclosure schemes are confirmed by the collaborative—filtering-based model and nudging prompt. Another approach [42] uses Hyperledger Fabric (HF) based consent management platform which is web-based. It uses HF’s Node TS SDK to interact with Hyperledger Fabric Network (HFN) from the front-end. In this platform, patients’ login into the platform to browse the available data consumer requests and makes choice of where to enrol. Platform also provided option to revoke and update provided consent. Framework uses chain code to ensure that only registered application can send a transaction to the ledger. Consentio [43] is a scalable consent management system based on the Hyperledger Fabric permissioned blockchain.it deals with individuals and their resources (data), data consumers and their roles, as well as watchdogs. To allow fine-grained consent specification, resources are divided into timeframes, with a time unit identified by time_id. There are four required consent management functionalities: Consent, Role, access request and audit. It addresses data management challenge to ensure high throughput and low latency of endorsing data access requests and granting or revoking consent. ADvoCATE [44] is a cloud service platform where new personal devices can be registered by providing the device name, serial number and type of device. The platform stores the provided information to the database using the pre-built schema, and the registered device is assigned by the platform a unique ID. After the successful registration of a new device, the vendor gets a notification about it with the corresponding device ID. The vendor’s id located at the serial number of the device, allows the platform to identify the corresponding vendor. The vendor creates a contract request with all the necessary data privacy information, such as processing purposes and recipients, and sends it to the user. The request is displayed on the user’s device, while the user’s response initiates the creation of an instance of a contract which will keep all the requested data and the user’s consent in a database entry. The source code of the smart contract is written in Solidity language and it is deployed to the Ethereum blockchain infrastructure per device. This contract manages all user’s consents for a specific device and can be updated or even withdrawn over time. More specifically, the platform supports four basic functions: the first one adds new consents (initial, updated or withdrawal) for a data controller, the second function returns the hash of the last consent for a data controller, the third returns the time that a specific consent was given to a data controller and the fourth function returns all the consents that are given to a specific data controller over time.

These approaches try to address the issues of consent handling and consent management using blockchain technology. However, these approaches do not deal with customer experience part about the tracking of sensitive data whether any data violations are happening and how regulatory bodies are actively involved into the whole eco-system. These approaches though provide enough reasons for using blockchain technology into the process of handling customer consent throughout its lifecycle.

### Blockchain Based Solution

**Fig. 1:**
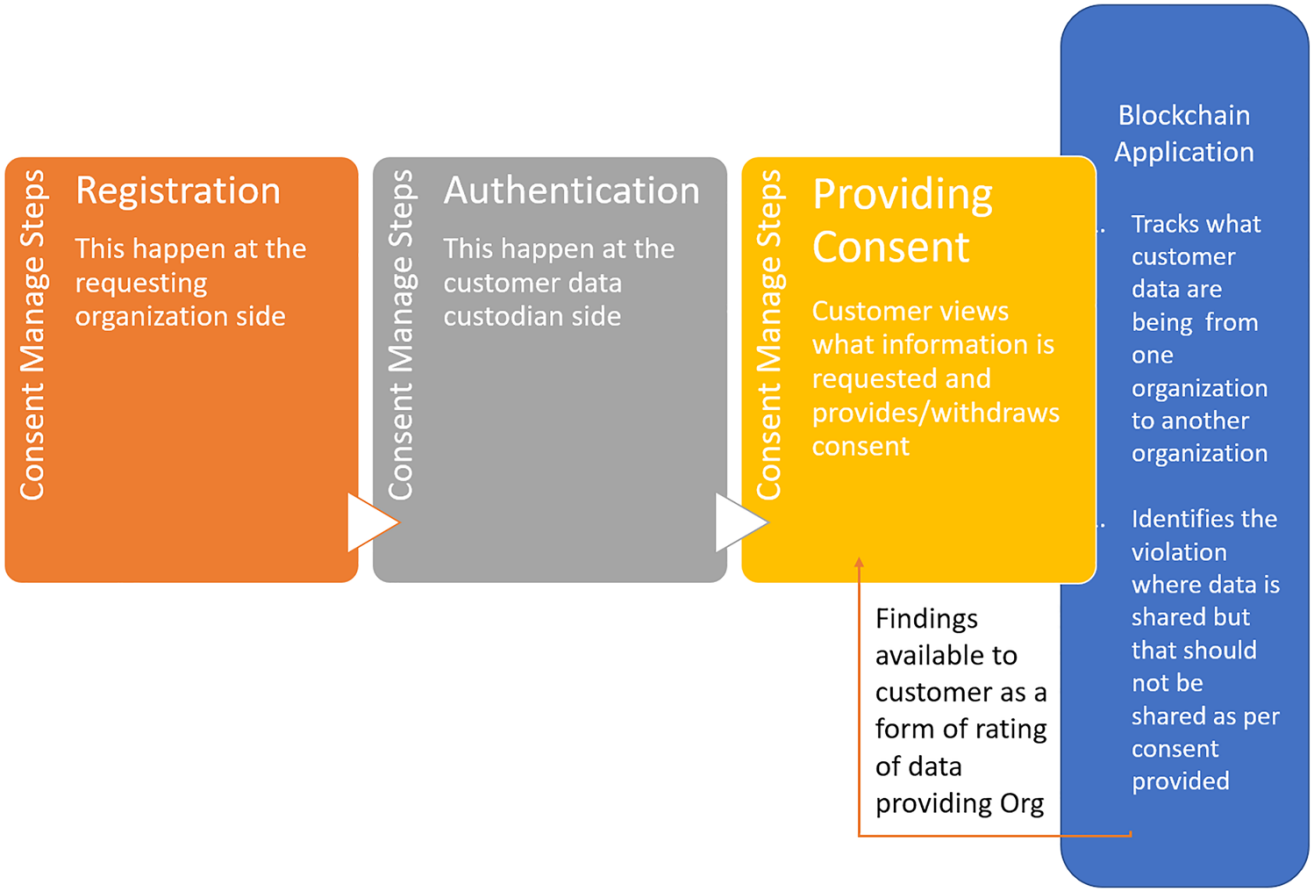
Blockchain application to effectively track data sharing violation

**Fig. 2:**
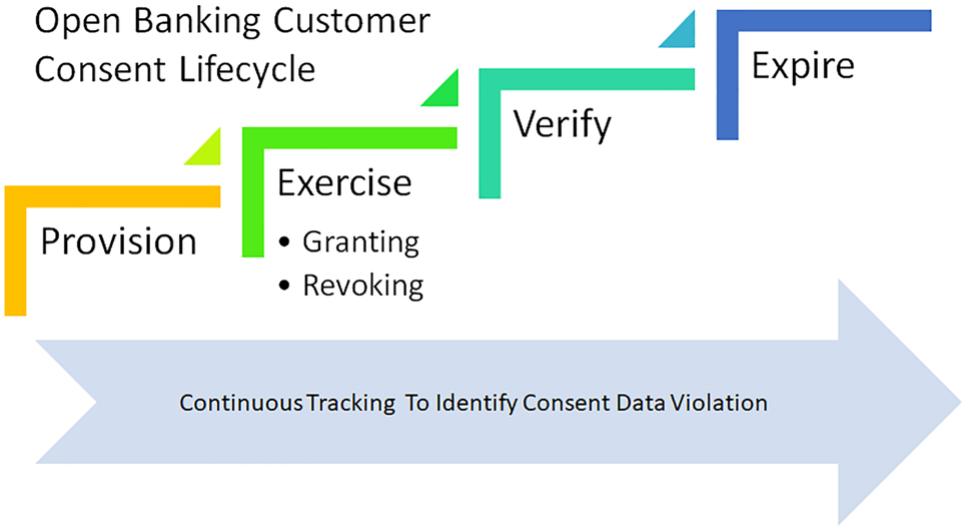
Open banking customer consent lifecycle

Kakarlapudi and Mahmoud [45] highlights that being compliant with GDPR rules are quite essential for Blockchain based solutions to boost overall participation from all parties. R. Dutta [46] analyses that while GDPR manages the policy side, blockchain can effectively manage the framework part. GDPR mandates that if consent provided by customer is revoked by customer then data needs to be deleted from shared parties. Blockchain fits perfect for managing consent as it is secured and cannot go into wrong hand and above all can be traced well. However sharing and deleting shared data needs consensus from involved parties. Consent management process does not need to have consensus from all parties in the consortium. It is essentially data sharing between two parties-from data custodian banks to the Third party providers. Consensus required between these two parties essentially whereas regulators need to be aware of the data sharing. The three steps of consent management- registration process, consent sharing and consent revoking need to have consensus in the consortium blockchain. Yao et al. [47] analyses the research challenges for consent management of the consortium blockchain which are Scalability enhancement, Algorithm combination, Privacy-preserving, Performance improvement, Searching and storing optimization. Based on our further analysis and research, we could identify some enterprise blockchain solution like Corda, Ethereum provides suitable solution to effectively manage most of these challenges. Based on our analysis on identified research gap, we can find out that feedback mechanism lacking in the processing steps which can help customers take informed decision. Figure 1 shows the sequential steps of customer journey in open banking. Existing processes and research areas focus on registration step, authentication step and providing consent step along with secured data handling. Not enough exploration happened on inducing information useful for customer taking decision on providing consent. Figure 2 elaborates the whole process and highlights additional step where one blockchain application is built which captures both the data- what data needs to be shared and what data actually have been shared. Comparing these two data sets, it identifies violation and informs customer promptly when such violation is happening. Customer can take prompt decision when such helpful information is provided to customers. As per regulations, customer can provide and withdraw consent at any point of time as per their wish.

This blockchain application can have multiple nodes and these nodes can be owned by participating organizations. Regulatory body can own the notary node to have tracking of every transaction.

## Implementation of Proposed Solution

Considering the importance of keeping customer informed, our approach is built on blockchain based framework for open banking consent tracking which can fit above any consent management system implementation. Framework tracks every data sharing from one organization (e.g. Bank A) to another organization (e.g. Bank B). Whenever data sharing happens, it is tracked as node to node transaction in corda implementation. It is explained in the sequence diagram of the framework how transaction initiated by customer in the TPP site, is notarized in the notary node which can be represented by the regulators. The framework shows that customer data handling between Bank and TPP are tracked as part of node to node transaction. Regulators will have the view of agreement between bank and TPP what customer data information can be shared as part of agreement. Any customer data information which is not part of agreement but shared with the TPP is tracked as a data violation case.

### Platform Design and Architecture

#### Requirements and Design Considerations.

The following section elaborates functional and non-functional requirements along with different design considerations:

##### Key Functional Requirements for Consent Tracking Platform.

As explained in Fig. 2, the lifespan of open banking customer consent involves different four stages which are provision, exercise, verify and expire. Effective tracking is required primarily in the exercise stage of the whole process as data sharing between bank and TPP essentially happens at this stage of data sharing.

Other than data sharing violation, tracking is required to ensure data is removed when customer is revoking the provided consent. Also an expired consent should not be considered as valid consent to share data with TPP. Our approach of effective tracking also involves about better customer experience where customer will be notified upfront in case of any data sharing violation.

##### Key Non-Functional Requirements for the Consent Tracking Platform.

The framework needs following criteria to be adhered in the adopted solution approach:

1. Regulator view: The secured transaction of data sharing happens between two participating parties but notary has the view of all transaction details. Regulators can own the notary node to have continuous tracking of data sharing.

2. Security: Permissioned consortium blockchain fulfills the demand of enhanced security in the network. It is not possible any unauthorized party to have a view in the shared data of customer.

3. Data retrieval and representation: Blockchain based approach provides this feature to retrieve the data securely. This helps in reporting data violations to customer directly from a regulatory body.

4. Compatibility: Framework can work along with any consent management system which makes it usable without any need to do any replacement of existing consent management system.

5. Scalability: It supports to go up to high number of nodes. For our working prototype, we have successfully tested with ten nodes and the numbers can go well above fifty and hundred. As a feature of corda, nodes can be added without making downtime for the running nodes.

#### Technical Architecture.

**Fig. 3:**
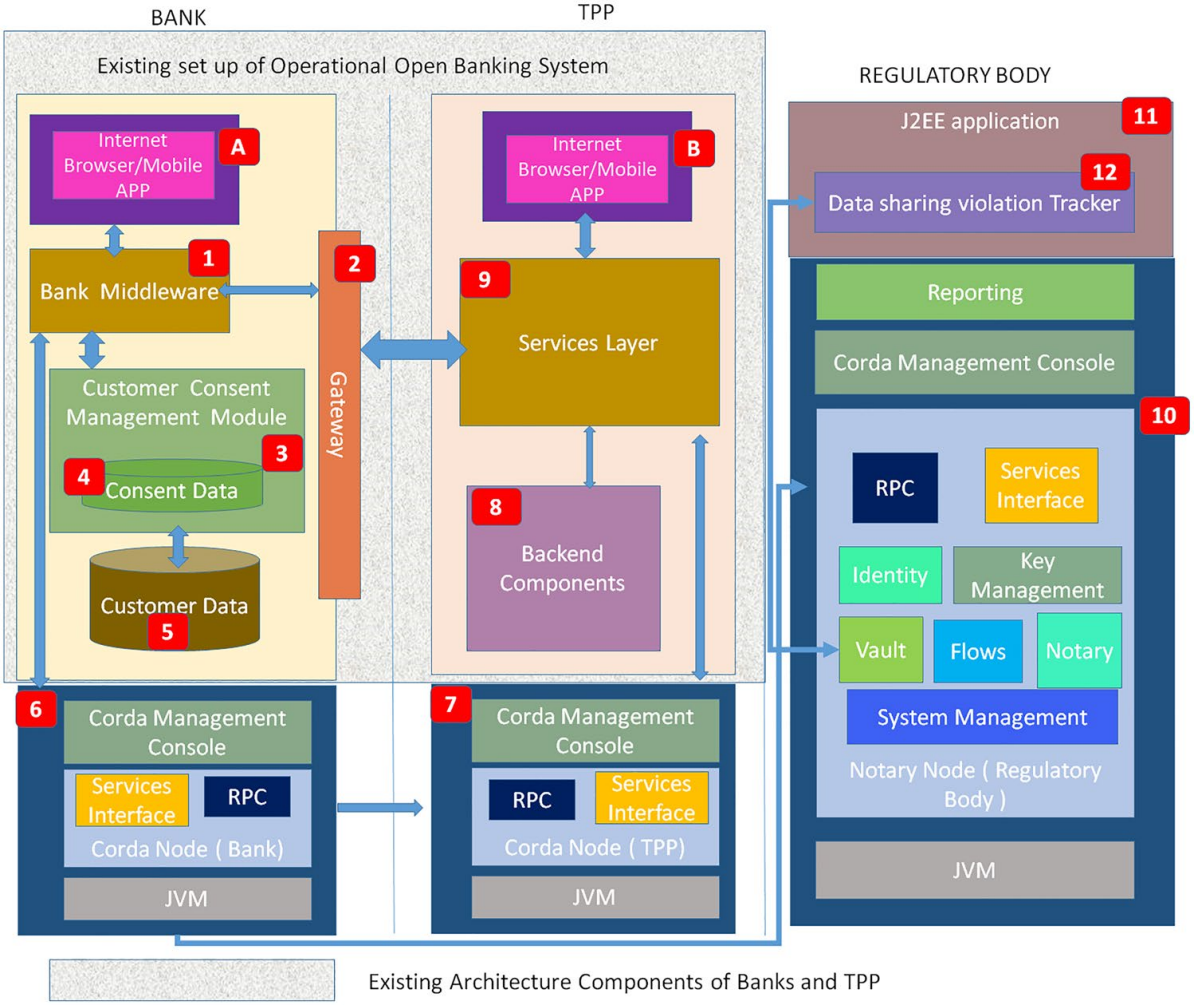
Technical Architecture of proposed solution

Figure 3 displays the proposed technical architecture highlighting the existing components in bank and TPP architecture along with new required components of framework. The diagram shows a part of it as existing set up of bank and TPP architecture. The new components are mainly required for regulatory bodies. Also bank and TPP need to set up blockchain nodes in their respective organizations. In this section it details out what are the components of the technical architecture and what main tasks are performed by these components. How these components interact each other and in which sequence, that is explained in the two sequence diagrams in Fig. 10 and Fig. 11.

(1) Key Terms

Following key terms are important for describing the framework:

- **Data Sharing Violation:** Customer data is shared between bank and TPP as per agreement or contract between bank and TPP. Customer has a view of it when customer registers for open banking services. This is not a fixed set of customer data types all the time and may vary depending on amendments in the agreement. Any data which is not mentioned in the agreement and which is not known to customer but that data is being shared between bank and TPP is to be considered as data sharing violation.
- **Consent Tracking:** Track the data sharing activity between bank and TPP by keeping record of what data being shared. These records are stored in the database of blockchain node which makes the data completely secured from mishandling.
- **Informed decision:** When customer providing consent of data sharing, at that time customer should be aware what happened to their data in the past when such data sharing happened. Was there any data sharing violation in the past? Such kind of information helps customer to take decision about providing data sharing consent.

(2) Key Components:

**Banking Digital channels:** Customer uses this channel to provide consent or revoke consent after successful authentication. It can be mobile app or internet banking channel which can be accessed by customer.

**TPP Digital channels:** Customer uses this channel to access the service provided by TPP. This channel redirects to TPP side services layer to redirect request to bank gateway for connecting to bank’s consent management module.

**Bank Middleware Component:** This component is responsible to accept call from TPP side via gateway and exposes API for TPP services layer to consume it for managing customer consent before providing open banking services to customer. It sends response with consent details to TPP services layer via bank gateway.

**Bank Gateway:** This component manages the request coming from TPP services layer to redirect the request to bank middleware. As well as it redirects the response coming from bank middleware towards TPP services layer.

**Bank Customer Consent Management Module:** This layer consists of multiple component which manages customer consent. It deals with viewing consent, editing consent, removing consent and retrieving consent whenever request received from bank middleware.

**Bank side customer consent storage:** It stores customer provided consents on the bank side. This is relational data base to persist consent provided by customer.

**Bank side customer data storage:** This is core banking system which stores customer demographic details, customer accounts and transactional details. In open banking transaction, customer data only retrieved from this storage to share with TPP. No update is done as part of the flow.

**Bank side Corda Node:** This is bank side corda node which will consist of all components of corda nodes including node database and service interfaces. All data sharing requests initiated by different customers will go through this single corda node. This node interacts with TPP corda node and Notary node only.

**TPP Services Layer:** This component is responsible to accept call from bank side gateway and consumes Bank API to manage customer consent before providing open banking services to customer. It receives response with consent details from bank via bank gateway.

**TPP Backend Components:** This layer stores customer provided consents shared by bank components. It also stores other transactional details which customer generates while accessing services provided by TPP.

**TPP side Corda node:** This is TPP side corda node which will consist of all components of corda nodes including node database and service interfaces. All data sharing response initiated by different customers will be received by this single corda node. This node interacts with bank corda node only.

**Regulatory Body side Corda node:** This is regulatory side corda node which will consist of all components of corda nodes including node database and service interfaces. All data sharing between bank corda node and TPP corda node will be registered in the database of this corda node.

**Regulatory Body side J2EE application:** This J2EE application manages multiple aspects like retrieving bank node to TPP node transaction data which are stored in notary node db. It also manages to send communication to customer based on the response received from data sharing violation tracker component.

**Regulatory Body side data sharing violation tracker component:** This component compares the data shared between bank node and TPP node based data sets retrieved from notary node database. These datasets include data what has been shared between bank and TPP and also data what should be shared as per agreement.

### Blockchain Implementation

**Fig. 4:**
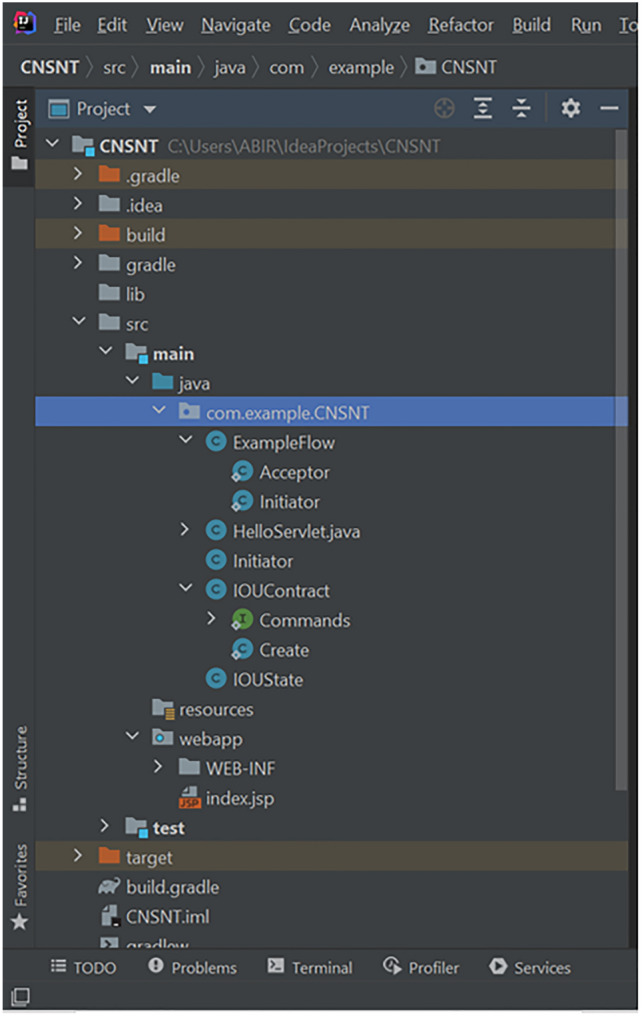
Blockchain implementation- structure of components

Corda blockchain implementation helps managing the required consensus among the participatory nodes. When bank needs to share data sharing contract details with regulatory body nodes, this transaction is not broadcasted to other participatory nodes. Regulatory body node being notary node, bank node can only send data sharing agreement with regulatory body owned blockchain node. In case of data sharing transaction, bank node shares data with TPP node and by virtue of corda blockchain implementation this transaction is broadcasted to notary node and verification is done about authenticity of this transaction. Even though there can be multiple notary node, for our implementation we have considered 1 notary node. We have used Glassfish 4.1 application server where bank existing architecture is replicated. This layer makes the RPC over AMQP call to connect to bank blockchain node. In sequence diagram, step 9b depicts this step. Figure 4 shows the blockchain implementation how it looks like the implementation structure.

**Fig. 5:**
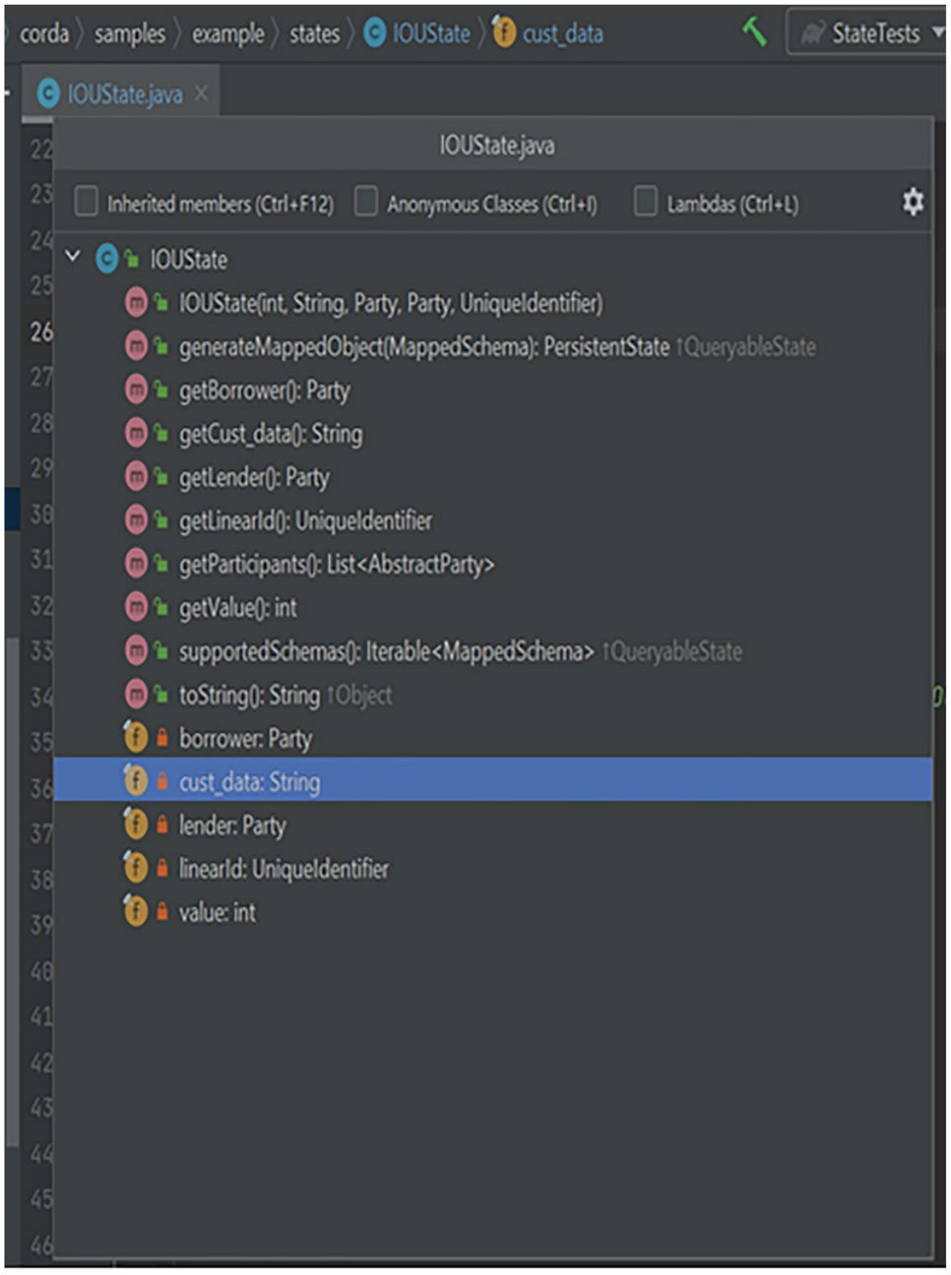
Component Structure of IOUState

**Fig. 6:**
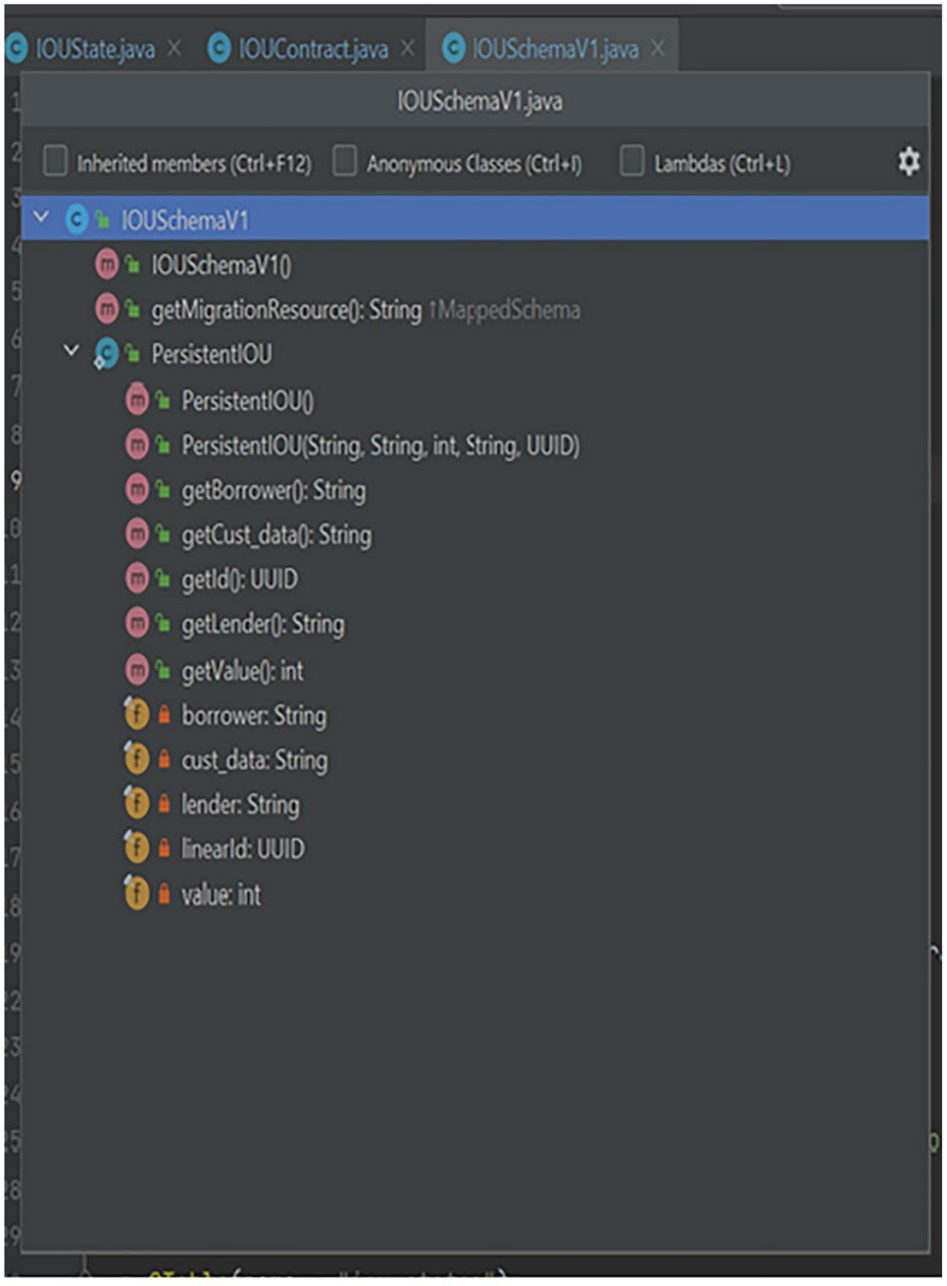
Component Structure of IOUSchema

**Fig. 7:**
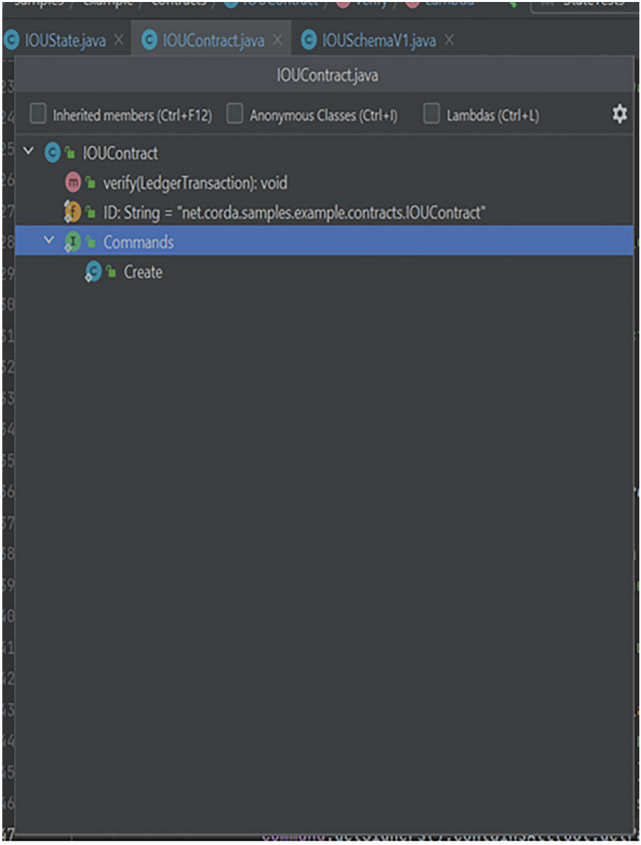
Component Structure of IOUContract

Figures 5, 6 and 7 shows the code structure of three key component in the blockchain implementation-IOUState.java, IOUContract.java and IOUSchema.java.

**Fig. 8:**
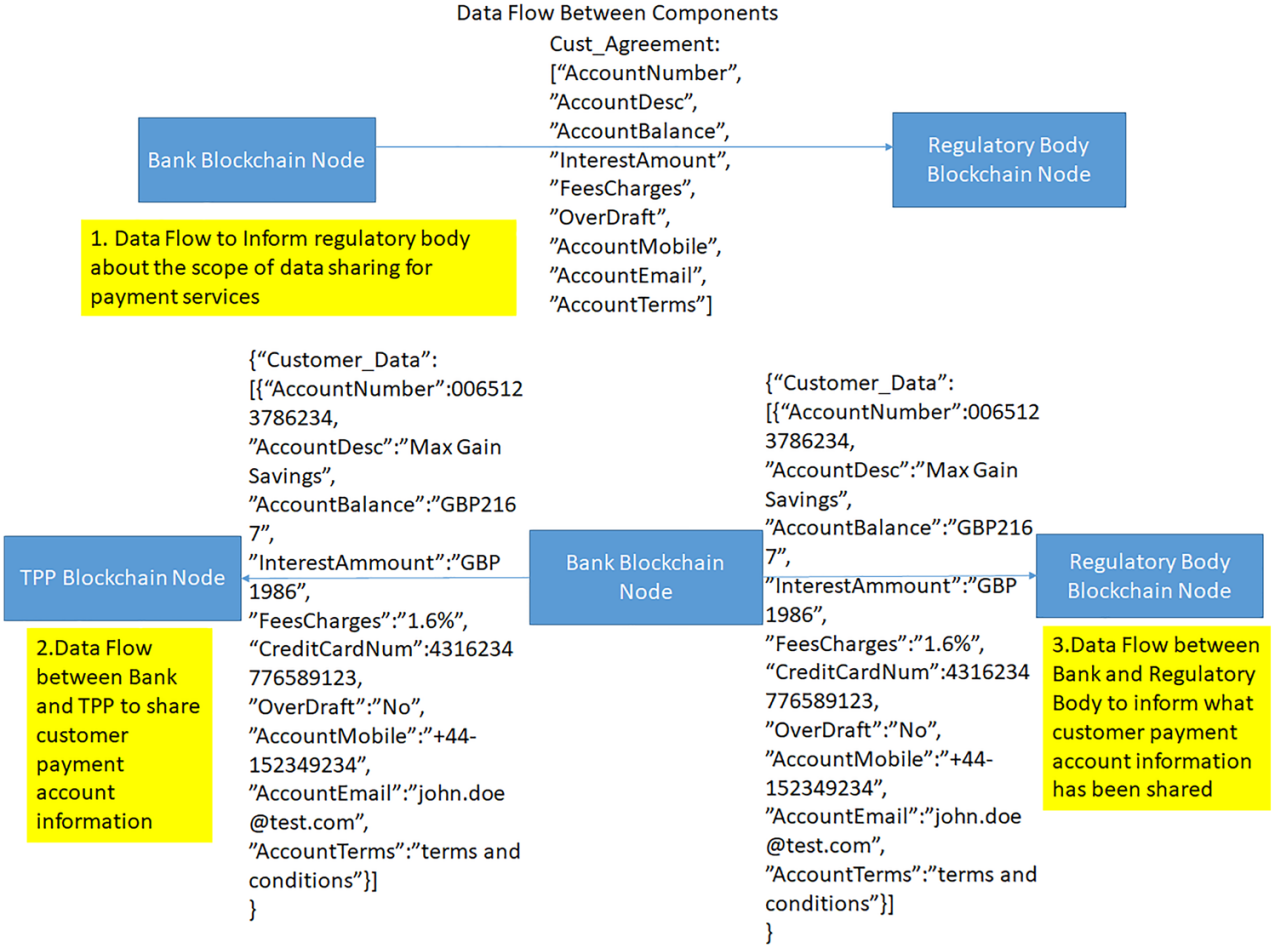
Data flow between blockchain nodes

These key components are critical to handle the customer data sharing transaction between nodes in our blockchain implementation in Corda. Figure 8 shows the shared data flow between nodes.

### Consent Tracking Process 

**Fig. 9:**
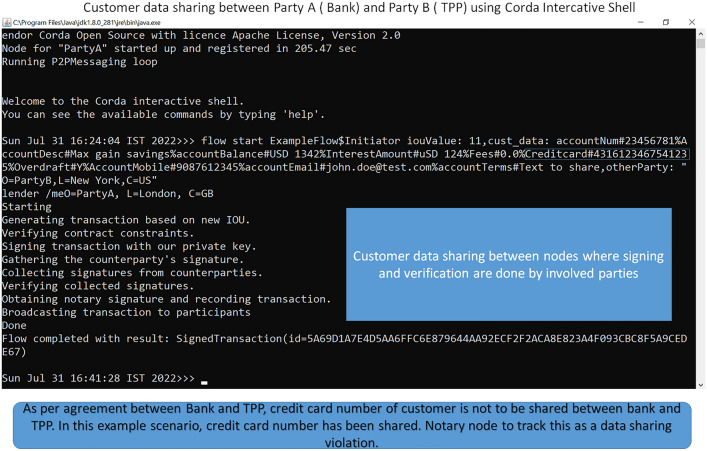
View of data sharing between bank and TPP using node to node transaction

This section describes the consent tracking process in sequential steps:

1. Customer accesses TPP site to get the required service/product
2. In case customer is not yet registered for open banking services, TPP will redirect request to bank to have the registration done. If customer is registered for open banking, customer consent status is checked. To check it, TPP makes a call through bank gateway. Bank receives the request and check customer consent status inside bank consent management module. This part of the flow and components are expected to be an existing set up in any operational open banking environment.
3. Bank acknowledges data access request from the TPP and initiates one node to node transaction through RPC call to notify the node owned by Notary what all data will be shared with the TPP as per the request.
  - Regulatory body stores which all data can be shared as per consent provided by customer
  - This blockchain based transaction part is additional which we are proposing as part of our framework
4. Bank sends authentication challenges to customer which needs to be successful for the data sharing with TPP. This is also expected to be an existing flow in any operational open banking system.
5. For successful authentication, Bank side blockchain node initiates another node to node transaction with TPP node through RPC call to share customer account/payment data with TPP. This is additional part in any existing open banking system which we are proposing as part of our suggested framework. View in Fig. 9 elaborates it.

5. TPP then reads shared data from Vault and allows customer to complete transaction using shared data from bank.
  - Regulatory Body has access to the shared data as notary node and compares what data has been shared with what all data can be shared as per consent. As shown in Fig. 4 data sharing view, credit card number of customer is shared violating data sharing agreement.
  - Regulatory Body informs customer if any extra data shared violating consent provided by customer
  - Customer gets notification if any data handling violation happened
6. If customer is happy that no data violation happened, customer completes the transaction at TPP site using shared data

**Fig. 10:**
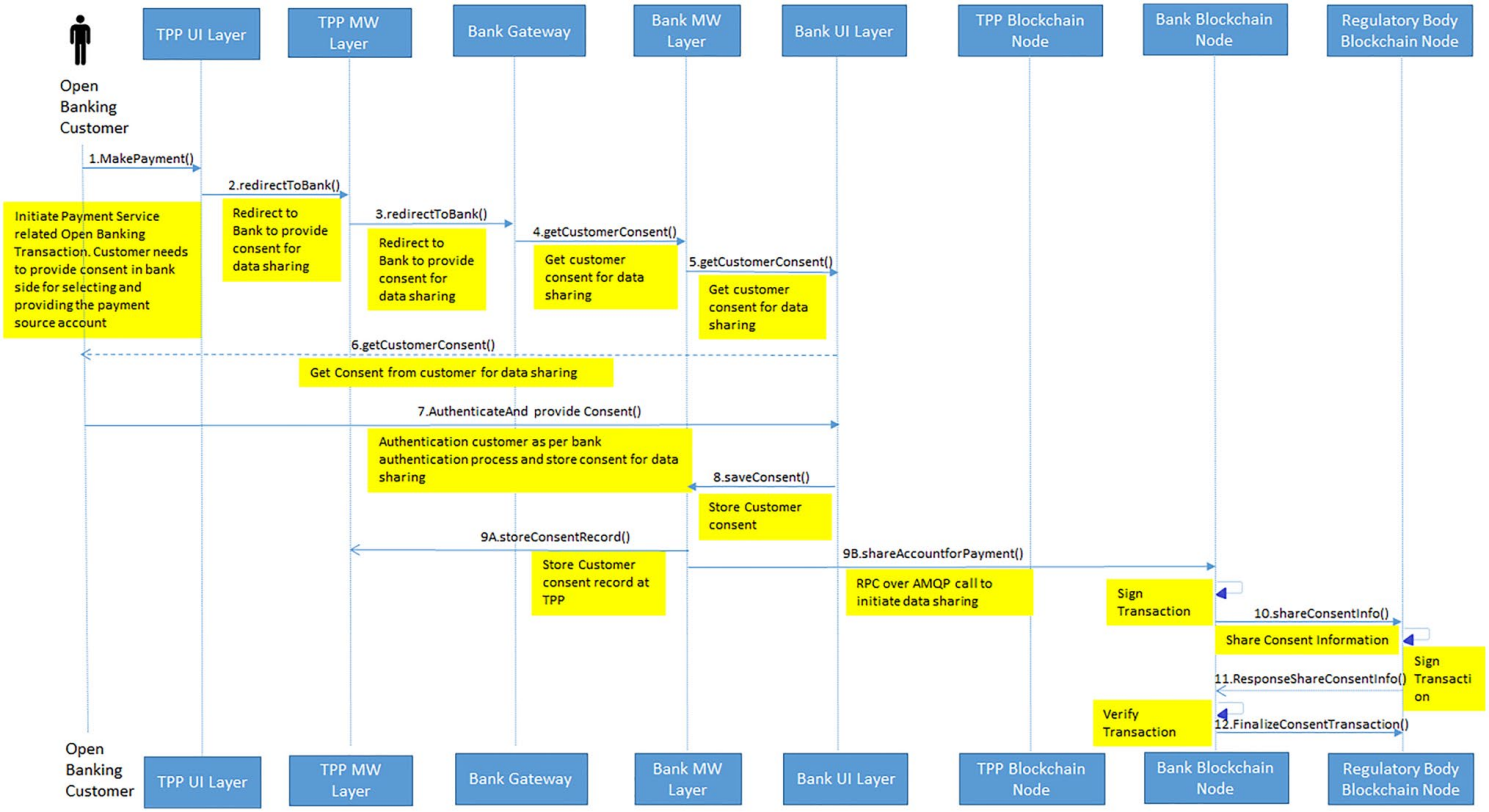
Sequence diagram of consent sharing in open banking transaction

**Fig. 11:**
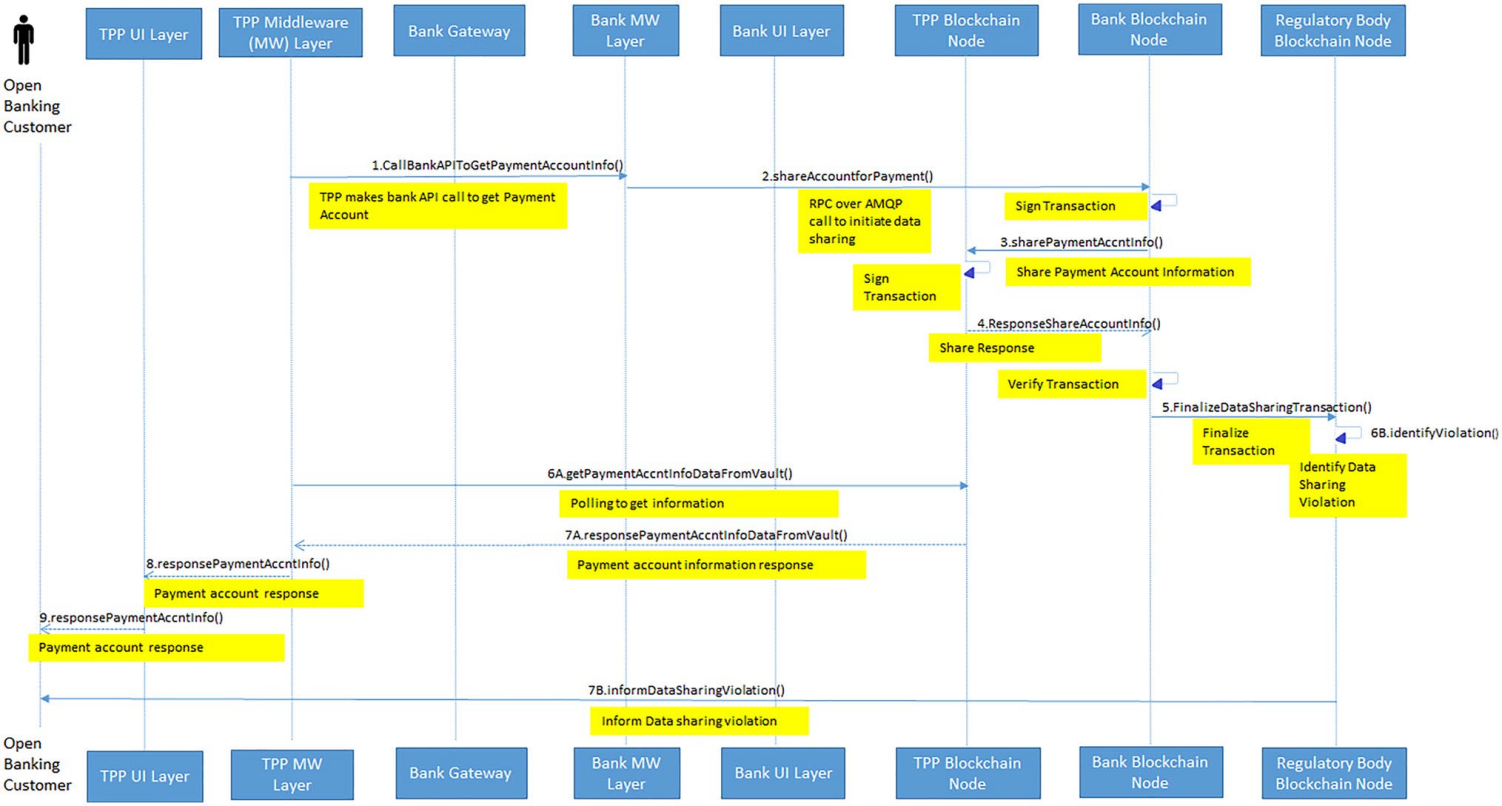
Sequence diagram of data sharing and violation tracking in open banking transaction

The sequence diagrams in the Figs. 10 and 11 provides the sequence of calls happening between the bank, TPP, regulatory node, bank node and TPP node. In the consent tracking sequence diagram it shows how request from customer is routed to bank through TPP middleware and it shows how bank node to notary node transaction happens to share data sharing contract details with regulatory body. Subsequently in the data sharing violation tracking diagram, it shows how data sharing happens between bank node and TPP node. As part of Corda node to node transaction, this transaction is also shared with regulatory body node.

**Fig. 12:**
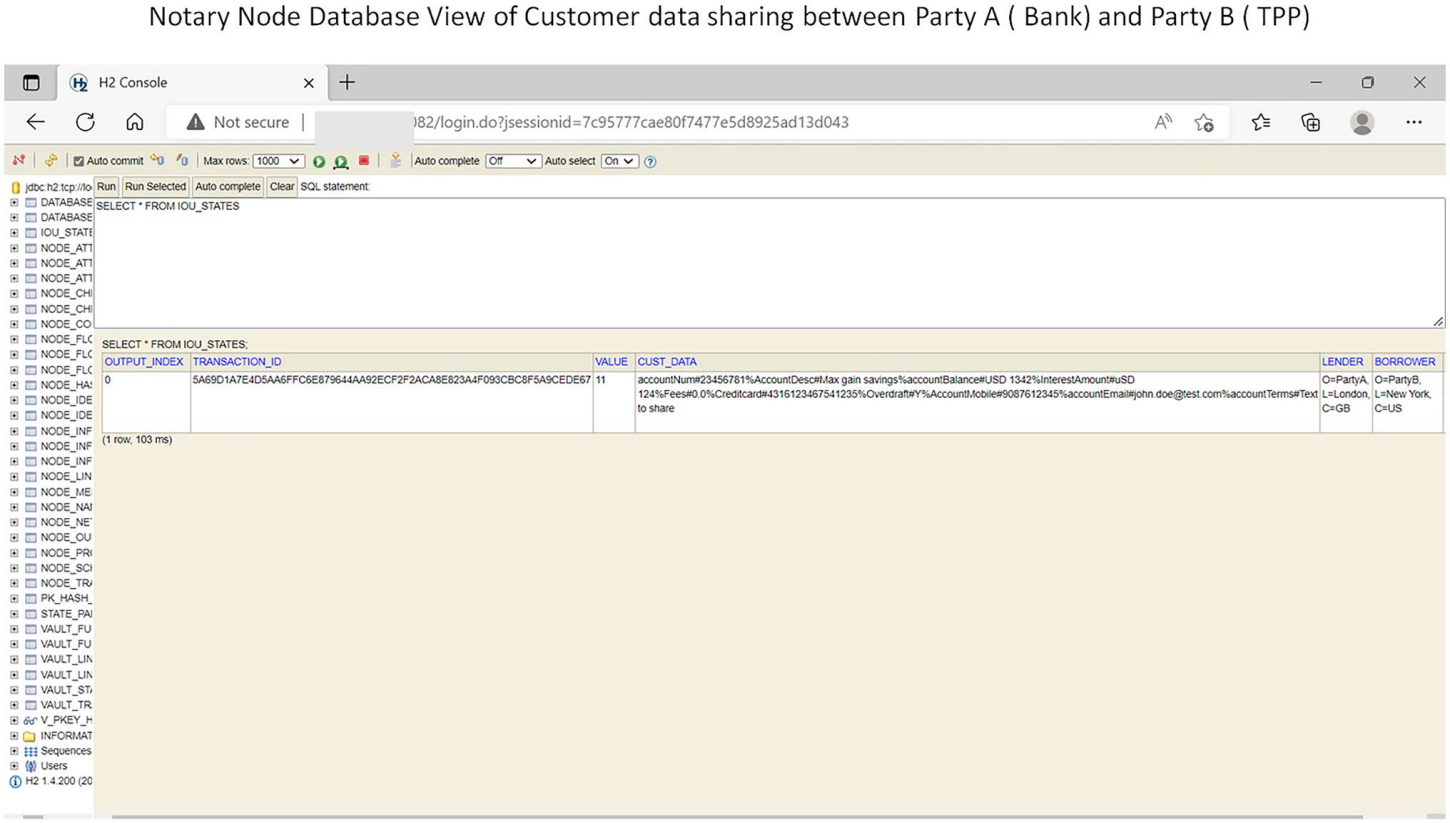
Notary node database view of bank node and TPP node transaction

In Fig. 12 notary node view is highlighted how regulatory body will access the transaction details between bank node and TPP node.

**Fig. 13:**
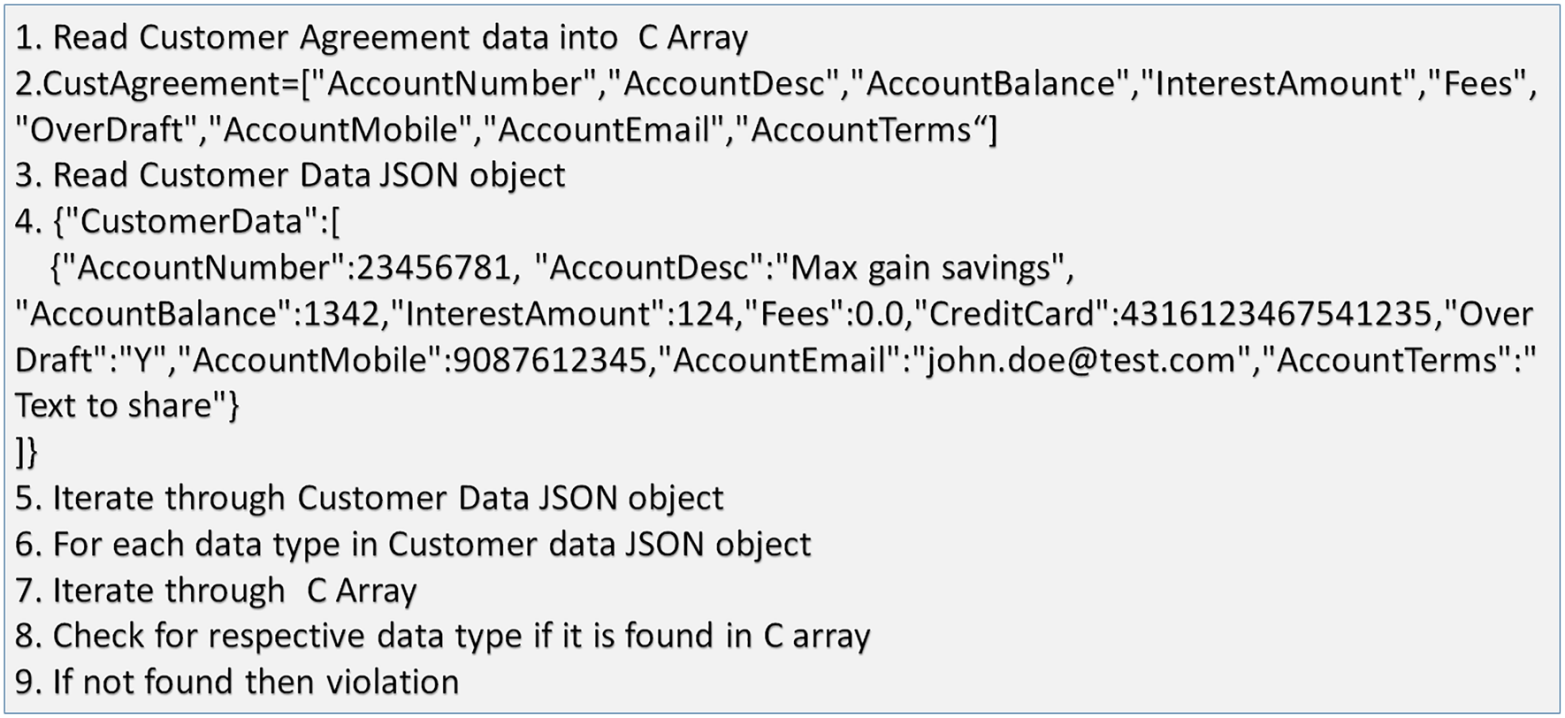
Data Violation Tracking Algorithm

In Fig. 13 algorithm shared how data violation tracking component identifies the data sharing violation. Though this tracking algorithm is straight forward and is simple data comparison between two sets of data, criticality of it lies in the fact that data sharing between bank and TPP cannot be altered by TPP or banks. In this data comparison, customer data will be set of data which has been shared with TPP. Data modification is not possible while reporting to regulatory body since blockchain implementation does not allow data distortion.

**Fig. 14:**
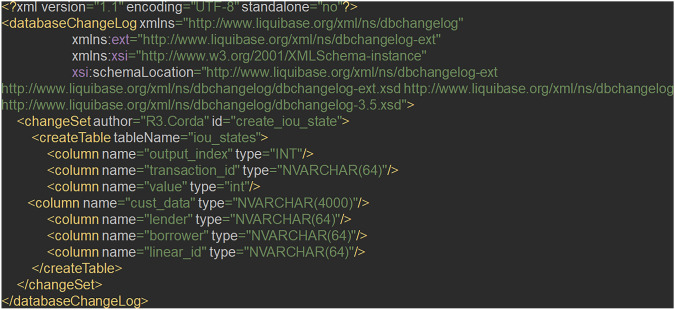
Node database modification

The code snippet in Fig. 14 shows the required modification done in node DB. This modification is required to pass the required number of customer data parameters to the target node.

### Communication, Security and Performance

**Fig. 15:**
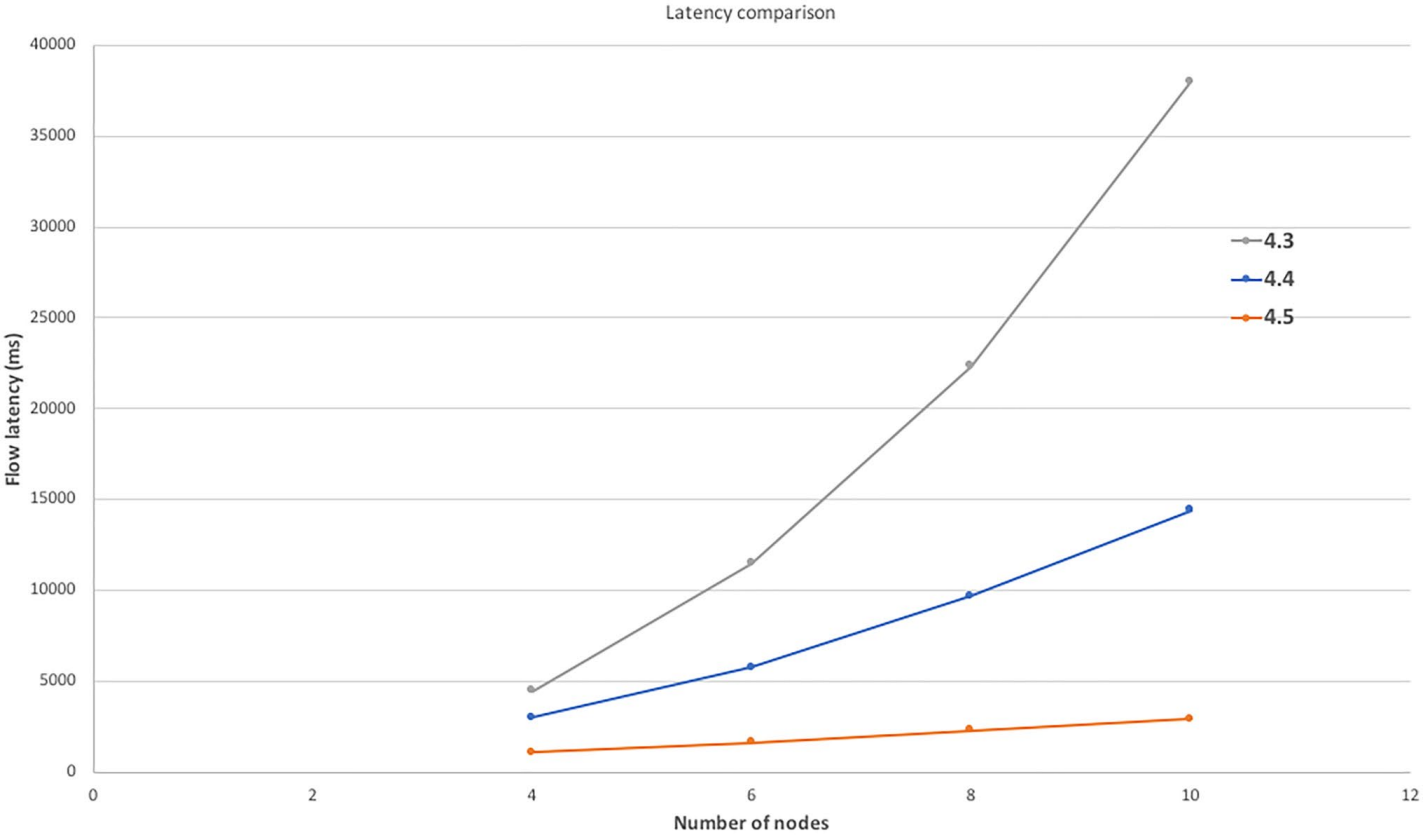
Flow Latency [48]

Connection between bank side Micro-services architecture components with corda node happens through RPC. For our proof of concept purpose, we did not go for message broking. However in a production environment with higher load, brokered messaging might be preferred option. In such cases asynchronous messaging will be a desired option. Specially the part where customer registration details of open banking are shared with notary node. Since we are suggesting corda based approach, it provides scalability, faster response time and transaction privacy on higher side than most of the available blockchain platform. Corda Enterprise 4 offers a substantial increase in Transactions per Second over the prior releases on the same hardware. Corda documentation [48] confirms about significant improvement in performance in the latest Corda Enterprise version. Figure 15 shows the improvement in handling the latency issue in recent Corda version.

We have tested our framework with number of customer data parameters ranging from 10 to 50 and we observed that there is no impact on the framework performance with the increase in the number of customer data parameter handling and number of nodes in the framework.

### Comparative View

In Table 1 a comparative view is elaborated where our blockchain framework is assessed with similar data privacy related implementation approach with blockchain based solution. Our solution is focused on open banking and very specifically consent handling and data sharing part of open banking whereas other solutions [41, 44] are generic in nature and does not address very specific problem of any industry. The most critical differentiation is in the nature of implementation where our approach is suitable to be fit on any existing banking technology landscape. All the other solutions [41–44] suggests to be built as standalone solution where the part remains unanswered is how banking like organizations deal with their existing technology landscape. Performance of Our blockchain framework is quite good and supports more than 10 nodes and supports more than 100 data parameters shared between bank and TPP in a single data sharing. In comparison, some solutions [41, 44] are designed to support high number of nodes but does not provide any indicative details. Our blockchain framework is built with the purpose of sharing a continuous access and view to one or many regulators about every data sharing transaction. Other solution [41–44] do not provide any specific indication of sharing access with regulators. Our blockchain framework uses validity consensus and unique consensus in comparison to other solutions by virtue of using the corda blockchain. It perfectly addresses our need as framework mainly deals with data sharing and does not deal with financial transaction.

**Table 1:** Comparative View of the Proposed Framework with similar implementation in blockchain

	ConsenTrack	O-Consent [41]	Data privacy management with Nudge Theory[42]	Enhancing User Privacy in IOT [43]	Consentio [44]
Application Area-Industry	Banking	Across Different Industries	Financial Sector	IOT Industry	Across Different Industries
Existing Architecture/Complete Overhaul	Existing Architecture	Complete Overhaul	Complete Overhaul	Complete Overhaul	Complete Overhaul
Implementation Coverage	Open Banking Customer Consent and Data sharing	Personal data processing	Data Privacy Management	The smart contracts User consent, GDPR-operation, Submission, and Verification	Consent Management System which can handles high throughput and low latency requirement
Blockchain Framework	Corda 4.6	Ethereum	Hyper ledger Fabric	Ethereum	Hyper ledger Fabric
Consensus	Validity Consensus and Unique Consensus	?	Utilized permissioned blockchain Hyperledger Fabric to manage consensus	?	Byzantine or crash fault tolerant consensus
Blockchain Type	Permissioned Consortium	Public Blockchain	Permissioned Blockchain	Public Blockchain	Permissioned Blockchain
Regulator View/Accessibility	Full Control to regulators	No real-time view by regulator	No real-time view by regulator	No real-time view by regulator	No real-time view by regulator
Customer Communication	Customer is communicated however customer interaction is not real-time	Customer involved in the process	Customer involved in the process	Customer involved in the process	Customer involved in the process
Secure data sharing platform	Yes	Yes	Yes	Yes	Yes
Secure Integrated service	Yes	Yes	Yes	Yes	Yes
GDPR compliant	Yes	Yes	Yes	Yes	Yes
Number of Nodes supported	Satisfactory test result with High Number of Nodes and low latency	Designed to have high number of nodes	Performance shared for 7 Nodes	Supports high number of actors( device)	Designed to have high number of nodes
Performance	Recent Enterprise edition of Corda shows significant improvement. It’s performance satisfactory with 10 nodes and more than 100 data parameters	Designed to address performance needs	High TPS	Verification cost increases and time for mining increases with increase in number of actors	Designed to have high TPS
Track TPP Access	Yes	Not applicable	No	Yes	Yes

### Issues and Challenges

Since regulatory bodies are very critical part of our solution approach, this needs to be approved and welcomed by the regulatory bodies. Every country has its own policies, priorities and strategies which are reflected in the open banking regulations framed by the regulatory bodies. So the acceptance or rejection chances and opportunities will vary from country to country. Need of infrastructure set up and governance will require regulatory body active participation into the whole process. There will be huge volume of data generation which can be effectively utilized by regulators to create insight for handling the future issues and customer concerns. It will also create necessity to frame regulations for taking prompt actions whenever any data sharing violation is identified in the data sharing transaction. Above all, TPPs and banking organizations need to be ready to accommodate the process of providing upfront information to customer. These organization also need to enhance their IT infrastructure little bit to support blockchain implementation which will work on top of existing technology landscape.

## Conclusion

Customer participation is not yet at desired level in Open Banking and one of the reasons is the fear in customer mind about the personal data being shared with undesired parties without being tracked. Keeping all these factors in mind, we have approached a solution which fits well on any existing open banking implementation. The solution suggests to use corda consortium blockchain for storing the registration and data sharing contract details. The framework approaches to store open banking consent registration data in the notary node database and this node is managed by regulatory authority. While doing any transaction, when customer data is shared by bank to TPP in the form of bank node to TPP node transaction, shared customer data is compared with stored consent registration data in the notary database. In case any data violation is tracked, such violation details are shared with customer instantly by the regulator. Corda blockchain support very high number of nodes without impacting the performance of system.

Information based transparent customer consent is essential to build trust about fair handling of open banking customer data in customer mind and also it is essential to remove the obstacles of dilemma in customer mind. Regulatory bodies need to own the notary node in this corda based blockchain implementation. The active participation of regulatory bodies in the whole process of consent management is absolutely needed to track the violation and sharing the violation with customer in real time. Regulatory bodies need to communicate customer as soon as any data sharing violation is tracked. Bringing this transparency into the data sharing process will boost customer confidence into the whole open banking products and services. Our research identifies this area as potential steer to have more participation from customer in open banking.

## Data Availability

The data that support the findings of this study are available from the corresponding author upon request.

## Abbreviations

TPP: Third party providers
API: Application programming interface
GDPR: General data protection regulation
IOT: Internet of Things
PSD 2: Payment Services Directive 2
HF: Hyperledger fabric
HFN: Hyperledger fabric network

## References

[1] Omarini A (2018). Banks and Fintechs: how to develop a digital open banking approach for the Bank’s Future. Int Bus Res.

[2] Official Journal of the European Union. REGULATION (EU) 2016/679 OF THE EUROPEAN PARLIAMENT AND OF THE COUNCIL of 27 April 2016. 2016. https://eur-lex.europa.eu/legal-content/EN/TXT/PDF/?uri=CELEX:32016R0679. Accessed 12 Oct 2022.

[3] Rantos, K, Drosatos, G, Ilioudis, C, Papanikolaou, A, Kritsas, A, Demertzis, K (2018). ADvoCATE: a consent management platform for personal data processing in the IoT using blockchain technology. 2018. Doi: 10.1007/978-3-030-12942-2_23.

[4] Stiefmueller C. Open Banking and PSD 2: the promise of transforming banking by ‘Empowering Customers’. 2020. 10.1007/978-3-030-51057-2_41.

[5] Solove DJ. Introduction: Privacy self-management and the consent dilemma. Harv Law Rev. 2012;126:1880.

[6] Bylykbashi S, Fitamant V, LEE J-Y. Consumers’ fears about open banking: How banks can overcome them?", http://archives.marketing-trends-congress.com/2021/pages/PDF/034.pdf. Accessed 29 Jan 2023.

[7] Bashir M, Hayes C, Lambert A, Kesan J (2015). Online privacy and informed consent: The dilemma of information asymmetry. Proc Assoc Inf Sci Technol.

[8] Athapaththu R. Consent management for open banking. 2019. https://wso2.com/ibrary/articles/2019/09/consent-management-for-open-banking/. Accessed 29 Jan 2023.

[9] Shafiq S. Consent Management: What You Need to Understand’. https://auth0.com/blog/what-you-need-to-understand-about-consent-management/. 2020. Accessed 29 Jan 2023.

[10] Miltiadou D, Soldatos J, Kyriazis D (2022). Leveraging management of customers’ consent exploiting the benefits of blockchain technology towards secure data sharing. Big data and artificial intelligence in digital finance.

[11] Babin R, Smith D (2022). Open banking and regulation: please advise the government. J Inf Technol Teachi Cases.

[12] ERI. Building Transparency with API/Open Banking. https://www.eri.ch/_include/WhitePapers/ERI-OpenBanking-Whitepaper.pdf. 2023. Accessed 29 Jan 2023.

[13] Remolina, N. Open Banking: Regulatory Challenges for a New Form of Financial Intermediation in a Data-Driven World (October 24, 2019). SMU Centre for AI & Data Governance Research Paper No. 2019/05, Available at SSRN: https://ssrn.com/abstract=3475019 or 10.2139/ssrn.3475019.

[14] Coiera E, Clarke R (2004). e-Consent: the design and implementation of consumer consent mechanisms in an electronic environment. J Am Med Inf Assoc JAMIA.

[15] Polasik M, Kotkowski R. The open banking adoption among consumers in Europe: The Role of Privacy, Trust, and Digital Financial Inclusion (April 30, 2022). Available at SSRN: https://ssrn.com/abstract=4105648 or 10.2139/ssrn.4105648.

[16] Martin K (2018). The penalty for privacy violations: how privacy violations impact trust online. J Bus Res.

[17] Rajaretnam T. The problem to consent to the collection, use, and disclosure of personal information in cyberspace. In: Proceedings Title: 2012 International Conference on Cyber Security, Cyber Warfare and Digital Forensic (CyberSec), 2012; pp. 283–288, doi: 10.1109/CyberSec.2012.6246124.

[18] Haksar V et al. Toward a global approach to data in the digital age. 2021. 10.5089/9781513599427.006.

[19] Mansfield-Devine S (2016). Open banking: opportunity and danger. Comput Fraud & Secur.

[20] Official Journal of the European Union (2016) REGULATION (EU) 2016/679 OF THE EUROPEAN PARLIAMENT AND OF THE COUNCIL of 27 April 2016. https://eur-lex.europa.eu/legal-content/EN/TXT/PDF/?uri=CELEX:32016R0679. Accessed 12 Oct 2022.

[21] Accenture. “PSD2 & Open Banking Security and Fraud Impacts on Banks Are You Ready?”. https://www.accenture.com/_acnmedia/pdf-40/accenture-psd2-open-banking-security-fraud-impacts.pdf. Accessed 29 Jan 2023).

[22] Leong E. Open Banking: The Changing Nature of Regulating Banking Data - A Case Study of Australia And Singapore (August 21, 2020). Banking & Finance Law Review, July 2020, Issue 35.3, pp 443–469, NUS Law Working Paper No. 2020/024, NUS Centre for Banking & Finance Law Working Paper 20/03, Available at SSRN: https://ssrn.com/abstract=367845. Accessed 29 Jan 2023

[23] Ozatac N, Saner T, Sen Z (2016). Customer satisfaction in the banking sector: the case of North Cyprus. Proc Econ Finance.

[24] Järvinen R (2014). Consumer trust in banking relationships in Europe. Int J Bank Mark.

[25] Wheatley S, Maillart T, Sornette D (2015). The extreme risk of personal data breaches & the erosion of privacy. Eur Phys J B.

[26] Whitley EA, Pujadas R. Report on a study of how consumers currently consent to share their financial data with a third party. 2018. https://www.fs-cp.org.uk/sites/default/files/fscp_report_on_how_consumers_currently_consent_to_share_their_data.pdf. Accessed 29 Jan 2023

[27] Joinson AN, Reips U-D, Buchanan T, Schofield CBP (2010). Privacy, trust, and self-disclosure online. Human-Comput Interact.

[28] Karwatzki S, Dytynko O, Trenz M, Veit D (2017). Beyond the personalization-privacy paradox: privacy valuation, transparency features, and service personalization. J Manag Inf Syst.

[29] Malhotra NK, Kim SS, Agarwal J (2004). Internet users’ information privacy concerns (IUIPC): the construct, the scale, and a causal model. Inf Syst Res.

[30] Metzger MJ (2004). Privacy, trust, and disclosure: exploring barriers to electronic commerce. J Comput-Mediated Commun.

[31] Sivathanu B (2019). An Empirical Study on the Intention to Use Open Banking in India. Inf Resour Manag J.

[32] Schlenker L. Identity, trust, and value(s): the future of Open Banking. https://towardsdatascience.com/identity-trust-and-value-s-the-future-of-open-banking-7926e22f085b. 2019. Accessed 29 Jan 2023.

[33] Farrell S. Banking on Data: a comparative critique of common-law open banking frame-works. 10.26190/unsworks/24096.

[34] Daiy AK, Shen K-Y, Huang J-Y, Lin TM-Y (2021). A hybrid MCDM model for evaluating open banking business partners. Mathematics.

[35] Alja P. Does the same word mean the same thing? An exploration of the notion of consent in PSD2 and GDPR. http://arno.uvt.nl/show.cgi?fid=148995. Accessed 29 Jan 2023.

[36] Esterik-Plasmeijer P, van Raaij F (2017). Banking system trust, bank trust, and bank loyalty. Int J Bank Mark.

[37] Sekhon H, Ennew C, Kharouf H, Devlin J (2014). Trustworthiness and trust: Influences and implications. J Mark Manag.

[38] Cheng L, Liu F, Yao D (2017). Enterprise data breach: causes, challenges, prevention, and future directions: Enterprise data breach. Wiley Interdiscipl Rev Data Min Knowl Discov.

[39] Mukhopadhyay I, Ghosh A. Blockchain-based framework for managing customer consent in open banking. In: The “Essence” of network security: an end-to-end panorama, Lecture Notes in Networks and Systems 163. 2021. 10.1007/978-981-15-9317-8.

[40] Mitra S. OConsent: open consent protocol for privacy and consent management with blockchain. 2021. 10.13140/RG.2.2.26751.12967.

[41] Ma S, Guo C, Wang H, Hong X, Xu B, Dai H-N, Cheng S, Yi R, Wang T (2018). Nudging data privacy management of open banking based on blockchain. 72–79. 10.1109/I-SPAN.2018.00021.

[42] Barati M, Rana O. Enhancing user privacy in IoT: INTEGRATION of GDPR and Blockchain (2020) 10.1007/978-981-15-2777-7_26.

[43] Agarwal R, Kumar D, Golab L, Keshav S (2019) Consentio: Managing Consent to Data Access Using Permissioned Blockchains. Proceedings of the 2020 IEEE International Conference on Blockchain and Cryptocurrency (ICBC), IEEE; Toronto, ON, Canada, pp. 1–9. Accessed 2 May 2020.

[44] Rantos K, Drosatos G, Ilioudis C, Papanikolaou A, Kritsas A, Demertzis K. ADvoCATE: a consent management platform for personal data processing in the IoT using blockchain technology. 2018. 10.1007/978-3-030-12942-2_23.

[45] Kakarlapudi P, Mahmoud Q (2021). A systematic review of blockchain for consent management. Healthcare.

[46] Dutta R, Das A, Dey A, Bhattacharya S. Blockchain vs GDPR in collaborative data governance. In: Cooperative design, visualization, and engineering (Lecture Notes in Computer Science), vol. 123410. Cham, Switzerland: Springer; 2020, pp. 81–92. 10.1007/978-3-030-60816-3_10

[47] Yao W, Ye J, Murimi R, Wang G (2021). A survey on consortium blockchain consensus mechanisms. arXiv:10.48550/arXiv.2102.12058

[48] R3. Corda Documentation. https://docs.r3.com. Accessed 29 Jan 2023.

